# Supplementary material for: Patterns of Thyroid Hormone Prescription in Patients with Bipolar or Schizoaffective Disorder: Findings from the LiSIE Retrospective Cohort Study
Source: J Clin Med. 2021 Oct 29;10(21):5062. doi: 10.3390/jcm10215062 (PMC8584539; doi:10.3390/jcm10215062)
Supplement: Supplementary file 1 [file jcm-10-05062-s001.zip › jcm-1399249-supplementary.pdf]

Supplementary Materials:

Table S1: STROBE Statement - checklist for our study

STROBE requirement	#	Our study
Title and abstract	1	
(a) Indicate the study's design with a commonly used term in the title and abstract		(a) Given: "Patterns of thyroid hormone prescription in patients with bipolar or schizoaffective disorder. Findings from the LiSIE retrospective cohort study"
(b) Provide in the abstract an informative and balanced summary of what was done and what was found		(b) Structured abstract provided.
Introduction		
Background/rationale: Explain the scientific background and rationale for the investigations being reported	2	Background outlined in introduction.
Objectives:<br>State specific objectives, including any pre-specified hypotheses	3	Aims clearly stated in text, "we sought to explore patterns of TRT use in patients with BD and SZD. Specifically, we tested the following three hypotheses:<br>1. In the majority of patients with BD/SZD, THRT is prescribed only for mild or no alterations of thyroid function tests (TFT) and/or unspecific symptoms.<br>2. The TSH concentration, at which THRT is initiated (TSH_{THRT}), has decreased over time.<br>3. In patients treated with lithium, TSH_{THRT} is lower compared to other MS.
Methods		
Study design:<br>Present key elements of the study design early in the paper	4	Study design: Retrospective cohort study.<br>Key elements of the study included in the manuscript: study design, participants, selection: inclusion and exclusion criteria, outcome definition, variable definitions, validation process, chart review, control for bias, missing data and statistical analysis.
Setting:<br>Describe the setting, locations, and relevant dates, including periods of recruitment, exposure, follow-up, and data collection	5	Setting and all relevant dates described in manuscript: "LiSIE invited all individuals in the Swedish regions of Västerbotten and Norrbotten of at least 18 years of age, who had either received a diagnosis of BD (ICD F31), schizoaffective disorder (SZD) (ICD F25), or who had used lithium as a mood stabiliser between 1997 and 2011."<br>"For the current study, we included patients from the region of Norrbotten who had received (a) a diagnosis of either BD or SZD on at least two occasions, at least six months apart any time between 1997 and 2013, (b) at least one THRT prescription (levothyroxine or liothyronine) between 1997 and 2017, and (c) their first THRT prescription after the diagnosis of BD/SZD, or after start of the MS treatment. For hypothesis 1, exploring TFT and thyroid-related symptoms at THRT initiation, we included the whole sample. For hypothesis 2, exploring TSH_{THRT} time trends, we used the sample for hypothesis 1, except patients who (a) had received THRT as augmentation therapy, or (b) had started THRT in relation to pregnancy. For hypothesis 3,

		examining TSH_{THRT} by MS, we used patients sampled for hypothesis 2, who had received MS treatment "
Participants: *(a)* Give the eligibility criteria, and the sources and methods of case ascertainment and control selection. Give the rationale for the choice of cases and controls (*b*) For matched studies, give matching criteria and the number of controls per case	6	(a) As above "The medical records of all eligible patients were retrospectively reviewed for the outcomes and variables under study, from 1 January 1997 up to 31 December 2017**."** (b) N/A.
Variables: Clearly define all outcomes, exposures, predictors, potential confounders, and effect modifiers. Give diagnostic criteria, if applicable	7	Definition for exposures and variables given in text. Outcomes stratified by gender, age, subtypes of bipolar disorder, lithium-use, mood stabiliser combination therapy compared to monotherapy, and mood stabiliser treatment stability.
Data sources /measurement: For each variable of interest, give sources of data and details of methods of assessment (measurement). Describe comparability of assessment methods if there is more than one group	8	Data source for all variables: electronic medical records. Definition for each variable given in text.
Bias: Describe any efforts to address potential sources of bias	9	Potential sources of bias discussed, including selection and observer bias. "We checked for selection bias in the whole retrospective cohort study (LiSIE). Age, sex, and where applicable, maximum recorded lithium and creatinine concentrations were key parameters, available in anonymised form. In accordance with the ethics approval granted, we compared these parameters for consenting and non-consenting patients. No significant difference was found between the two groups."
Study Size: Explain how the study size was arrived at	10	Cf. figure 2 "Of 1564 included patients with BD or SZD, 421 (26.9%) had received THRT at some point. Of these, 359 patients started THRT within our review period. A total of 291 patients met our inclusion criteria (Figure 1). For hypothesis 1, we included all 291 patients. For hypothesis 2, we included a subset of 281 patients. For hypothesis 3, we included a subset of 260 patients (Table 2)."
Quantitative variables: Explain how quantitative variables were handled in the analyses. If applicable, describe which groupings were chosen and why	11	Main outcome: Outcome summarized in the following categories: (1) Thyroid status at which THRT was started based on thyroid function tests, (2) Reasons for THRT initiation (3) TSH at THRT initiation over time, (4) Time from starting MS to starting THRT Handling of variables described in statistical methods as below

Statistical methods:<br>*a)* Describe all statistical methods, including those used to control for confounding<br>*(b)* Describe any methods used to examine subgroups and interactions<br>(*c)* Explain how missing data were addressed<br>*(d)* If applicable, explain how matching of cases and controls was addressed<br>*(e)* Describe any sensitivity analyses	12	(a) All data were anonymised before statistical analysis. We first analysed the data descriptively, with means and medians for continuous variables and frequencies for categorical variables. For the analysis of relations between categorical variables, chi-square tests were used. Two-sided Fisher's exact test was used if a table cell had an expected count of <5. We used z-test for two proportions to compare the sex distribution of the whole LiSIE cohort with the sex distribution for our sample. For each year under study, we performed time trend analyses for median TSH with quantile regression….. We used Kaplan-Meier plots to map the time from starting MS to starting THRT. (b) We used Mann Whitney U test to determine any potential differences in median TSH_{THRT} concentrations and median $fT4_{THRT}$ concentrations according to (a) diagnosis, (b) age category, (c) sex and (d) MS category. We used Kaplan-Meier plots to map the time from starting MS to starting THRT. The difference in these curves according to (a) lithium exposure, (b) combination therapy and (c) treatment stability, was analysed with a log-rank test. Throughout, the significance was set to p= 0.05.<br>(c) In terms of reasons for THRT initiation, we extracted all available information for the whole sample. When reasons were not documented, we specified this in the results (Figure 2). For two patients, case records were incomplete, and THRT prescription could not be validated.<br>(d) N/A<br>(e) N/A
Results		
Participants:<br>*(a)* Report numbers of individuals at each stage of study—eg numbers potentially eligible, examined for eligibility, confirmed eligible, included in the study, completing follow-up, and analyzed<br>*(b)* Give reasons for non-participation at each stage<br>*(c)* Consider use of a flow diagram	13	(a+b) Of 1564 included patients with BD or SZD, 421 (26.9%) had received THRT at some point. Of these, 359 patients started THRT within our review period. A total of 291 patients met our inclusion criteria (Figure 1). For hypothesis 1, we included all 291 patients. For hypothesis 2, we included a subset of 281 patients. For hypothesis 3, we included a subset of 260 patients (Table 2).<br><br>(c) Flow diagram included in the manuscript as figure 1.
Descriptive data:<br>*(a)* Give characteristics of study participants (e.g. demographic, clinical, social) and information on exposures and potential confounders<br>*(b)* Indicate number of participants with missing data for each variable of interest	14	(a) Baseline characteristics described in table 2 of the manuscript.<br><br>(b) Included in the flow diagram and in the text.
Outcome data:<br>Report numbers in each exposure category, or summary measures of exposure	15	Outcome data presented in text, in table 3 and figures 3-6
Main results	16	

(a) Give unadjusted estimates and, if applicable, confounder-adjusted estimates and their precision (eg, 95% confidence interval). Make clear which confounders were adjusted for and why they were included *(b)* Report category boundaries when continuous variables were categorized *(c)* If relevant, consider translating estimates of relative risk into absolute risk for a meaningful time period		(a) Results presented according to the statistical method outlined in item 12<br>(b) Results presented according to the statistical method outlined in item 12. Variable definitions given in method.<br>(c) N/A
Other analysis: Report other analyses done—e.g. analyses of subgroups and interactions, and sensitivity analyses	17	Sub-analysis according to the three hypotheses, cf. item 13
Discussion		
Key results: Summarize key results with reference to study objectives	18	Done
Limitations: Discuss limitations of the study, taking into account sources of potential bias or imprecision. Discuss both direction and magnitude of any potential bias	19	Limitation discussed in regard to selection bias, data quality, and potential for observer bias/recording error.<br>Other psychotropic medications<br>We also checked for other psychotropic medication that could affect thyroid function, such as tricyclic antidepressants (TCA) and phenothiazines.
Interpretation: Give a cautious overall interpretation of results considering objectives, limitations, multiplicity of analyses, results from similar studies, and other relevant evidence	20	Results discussed in view of the limitations (weaknesses) of our study design. Advantages and disadvantages of studies based on medical records compared to register studies discussed.
Generalisability: Discuss the generalizability (external validity) of the study results	21	Discussed in the context of bias. The sample under study is judged to be representative and the largest sample available for the topic under study.
Funding: Give the source of funding and the role of the funders for the present study and, if applicable, for the original study on which the present article is based	22	This work was supported by a grant of the Research & Development Fund of Norrbotten Region, Sweden.<br>Conflict to interest statement for all authors included in manuscript.

Source: http://www.strobe-statement.org/. Accessed 12 March 2019
